# Exploring the OncoGenomic Landscape of cancer

**DOI:** 10.1186/s13073-018-0571-0

**Published:** 2018-08-03

**Authors:** Lidia Mateo, Oriol Guitart-Pla, Miquel Duran-Frigola, Patrick Aloy

**Affiliations:** 1 Joint IRB-BSC-CRG Program in Computational Biology, Institute for Research in Biomedicine (IRB Barcelona), The Barcelona Institute of Science and Technology, Barcelona, Catalonia Spain; 2 Institució Catalana de Recerca i Estudis Avançats (ICREA), Barcelona, Catalonia Spain

## Abstract

**Background:**

The widespread incorporation of next-generation sequencing into clinical oncology has yielded an unprecedented amount of molecular data from thousands of patients. A main current challenge is to find out reliable ways to extrapolate results from one group of patients to another and to bring rationale to individual cases in the light of what is known from the cohorts.

**Results:**

We present *OncoGenomic Landscapes*, a framework to analyze and display thousands of cancer genomic profiles in a 2D space. Our tool allows users to rapidly assess the heterogeneity of large cohorts, enabling the comparison to other groups of patients, and using driver genes as landmarks to aid in the interpretation of the landscapes. In our web-server, we also offer the possibility of mapping new samples and cohorts onto 22 predefined landscapes related to cancer cell line panels, organoids, patient-derived xenografts, and clinical tumor samples.

**Conclusions:**

Contextualizing individual subjects in a more general landscape of human cancer is a valuable aid for basic researchers and clinical oncologists trying to identify treatment opportunities, maybe yet unapproved, for patients that ran out of standard therapeutic options. The web-server can be accessed at https://oglandscapes.irbbarcelona.org/.

**Electronic supplementary material:**

The online version of this article (10.1186/s13073-018-0571-0) contains supplementary material, which is available to authorized users.

## Background

The widespread incorporation of next-generation sequencing into clinical oncology has yielded an unprecedented amount of molecular data from thousands of patients, holding promise for a healthcare revolution [1, 2]. One of the current challenges is to find out reliable ways to extrapolate results from one group of patients to another and to bring rationale to individual patients in the light of what is known from the cohorts. In this context, visualization tools that enable the exploration and analysis of large genomic datasets become essential for efficient interpretation and effective communication.

Conventional strategies often represent dysregulated genes in a cohort as a matrix, with samples as columns and genes as rows, sorted according to the frequency of the genomic alterations [3–6]. Although this representation is useful to identify the main driver genes and to find recurrent patterns, it often misses the capability of capturing the global structure of a cohort of patients or the comparison to other cohorts. Other approaches are more focused on exploiting population structure patterns based on genomic profile similarities computed considering the whole genome or transcriptome [7–9]. The representations generated by these methods are difficult to interpret from a biological point of view since most of the genomic alterations considered are of unknown functional impact. Furthermore, with the exception of the recently presented TumorMap [7], the available tools do not offer a means to locate individual patient data within the cohort as a whole. In this context, as a complementary approach, we have developed a visualization tool to allow the global characterization of cohorts, and that focuses on driver alterations with known functional impact on oncogenesis, yielding a global picture of a cohort that is biologically interpretable.

## Implementation

### Dataset summary

We collected 16,508 genomic profiles (coding somatic mutations and copy number variants) that are representatives of several cohorts of patients and cancer models (patient-derived xenografts, organoids, and cell lines). We considered the 92.15% of samples having a putatively oncogenic alteration in one or more genes covered by the IMPACT410 gene panel (see Table 1). If solicited, future updates can easily incorporate larger patient cohorts, such as the complete TCGA [10] and ICGC [11] sets, and whole exome sequencing data, to complement the 410 genes included in the IMPACT panel [12].

**Table 1:** Summary of sample size and provenance

	Biological source	No. of samples with SNV and CNV data	No. of samples with driver alterations in whole exome	No. of samples with driver alterations in IMPACT410
TCGA [10]	Patients	4058	3935	3850
MSK-IMPACT [12]	Patients	10,945	–	9869
Novartis PDXs [17]	PDXs	375	375	375
OncoTrack [19]	Patients	117	109	109
	PDXs	59	59	59
	Organoids	46	46	46
GDSC Cell Lines [18]	Cell lines	908	904	904
TOTAL	–	16,508	5428	15,212

### Variant filtering

In order to filter out as many passenger alterations as possible, we applied a strict filtering pipeline described below, which was slightly tailored to each dataset:

#### TCGA patients

We downloaded the *Catalog of Driver Mutations – 2016.5*, a curated dataset of known and predicted oncogenic coding mutations identified after analyzing 6792 exomes of a PanCancer cohort of 28 tumor types [13]. We could complement this information with copy number variation data [14, 15] for 4058 patients, representing 16 tumor types. In addition to the known and predicted oncogenic coding mutations, we also considered as oncogenic the deletion (GISTIC score ≤ − 2) of tumor suppressor genes and the amplification (GISTIC score ≥ 2) of oncogenes. The role of driver genes was established by inspecting the *Catalog of Cancer* Genes [16].

#### MSKCC patients

We obtained both protein coding mutations (msk_impact_2017_mutations) and copy number variants (msk_impact_2017_cna) from the MSK_IMPACT Clinical Sequencing Cohort [12] through cBioPortal [14, 15]. Genes with a copy number alteration score ≤ − 2 or ≥ 2 were considered as putative deletions or amplifications, respectively.

#### Novartis PDXs

We collected the 375 PDXs for which both mutations and copy number alterations were available [17]. After analyzing the probability distribution of the estimated absolute copy number per gene, we considered absolute copy numbers below 1 or above 4 as gene deletions or amplifications, respectively. Using these criteria, we observed significant differences in gene expression between deleted tumor suppressors and amplified oncogenes (Additional file 1: Figure S1A), confirming that those thresholds are biologically relevant.

#### GDSC cell lines

We used gene level copy number data reported in the Genomics Drug Sensitivity in Cancer (GDSC) resource [18], which is based on PICNIC analysis of Affymetrix SNP6.0 arrays. We considered genes with a minimum copy number of any genomic segment mapping to that gene below 1 or above 6 as gene deletions or amplifications, respectively. Using those thresholds, we observed significant differences in gene expression between deleted tumor suppressors and amplified oncogenes (Additional file 1: Figure S1B), as described above for the analysis of copy number variants in PDXs.

#### OncoTrack [19]

We downloaded the genomic profiles of a biobank of 106 tumors, 35 organoids, and 59 xenografts. Copy number alterations were already annotated as “Amplification” or “Deletion.”

For MSK-IMPACT, Novartis PDXs, GDSC cell lines and OncoTrack datasets, protein-coding somatic mutations (following HGVS nomenclature recommendations), and copy number variants were classified into predicted passenger or known/predicted oncogenic alterations using the cancer genome interpreter resource [16].

After filtering out putative passenger alterations, we subsampled the dataset to consider only oncogenic alterations covered by the IMPACT410 gene panel [20], which provided a much larger reference cohort (> 10,000 patients MSKCC [12]) while retaining enough signal to build meaningful OncoGenomic Landscapes.

### 2D projections

We built a Boolean matrix encoding the oncogenic alterations identified in each sample (in rows) and driver gene (in columns). We then calculated the Jaccard distance between all pairs of unique samples and used the resulting distance matrix as input for a metric multidimensional scaling (MDS), carried out using the *scikit-learn* implementation of MDS [21] with default parameters (2 components, 4 SMACOF initializations, and a maximum of 300 iterations per run). As a result, we obtained (*x*, *y*) coordinates for each of the samples (i.e., a 2D projection). The corresponding level plots were generated by the 2D kernel density estimate function of the *seaborn* library, using 20 levels and a gray scale color-map as background. The PanCancer and more specific landscapes are the result of applying this procedure to the whole dataset and sample subsets, respectively.

To assess the significance of the distance metric and the dimensionality reduction strategy used to generate the landscapes, we examined whether the organization of samples in the PanCancer Landscape reflects the tissue-of-origin of the tumor. We observed a significant clustering of samples based on tissue-of-origin when examining both the Jaccard similarity coefficient in the multidimensional space and the Euclidean proximity in the MDS space. To evaluate the robustness of the current strategy, we also assessed the clustering of samples when using a Kernel PCA projection, an approach previously used in the field [9]. We observed that the MDS projection yields greater spatial resolution compared to Kernel PCA and that the proximity in the MDS space has a stronger correlation with the proximity in the multidimensional space (Additional file 1: Figure S2).

When new samples are to be mapped onto a given landscape, we approximate their location by a nearest neighbor search in the original multidimensional space of genomic alterations (i.e., Jaccard distance). A new sample is assigned the (*x*, *y*) coordinate of its nearest neighbor, and the distance between them serves as a confidence score of the mapping. We found this simple strategy to be sufficient, as it yields an error comparable to the intrinsic one of SMACOF MDS (Additional file 1: Figure S3).

### Cohort overlays

In order to highlight the territory occupied by a subset of samples, we obtained the (*x, y*) coordinates of the selected samples in a given landscape and generated a 2D kernel density estimate with the *kdeplot* function using 20 levels, a transparent background, and contours colored using a color-map that represents probability density as heat.

### Driver landmark overlays

Similarly, to highlight the territory occupied by samples that have an oncogenic alteration in a given driver gene, we obtained the coordinates of those samples and generated a 2D kernel density estimate using 4 levels. We modified the resulting plots by removing the level with the lowest density and setting the same color and transparency to the rest of levels.

### Survival analysis

We used the median distance to the 22 nearest PDXs, which correspond to 5% of the 434 Novartis PDXs, as a measure of how far a patient is to the PDXs. Patients in the upper and lower quartiles of the median distance distribution were considered to be distal or proximal to PDXs, respectively. We compared the lifespans of patients that are proximal or distal to PDXs using the Kaplan-Meyer estimate of the survival function and performed a log-rank test to assess the statistical significance of the observed difference using the *lifelines* library. Additionally, we investigated the effect of distance to PDXs on survival using Cox’s proportional hazards regression model, adjusting for tumor type and patient provenance covariates.

## Results and discussion

We have developed a visualization tool that is mainly focused on the global characterization of cancer cohorts. Our computational pipeline mines and integrates genomic profiles from 13,827 cancer patients and 1385 cancer models (434 patient-derived xenografts, 46 organoids, and 905 cell lines), compares pairs of samples based on shared oncogenic alterations, and plots the results in a 2D space that we called OncoGenomic Landscape. We offer our tool as a web-based interface that enables the comparison of the main cohorts published to date, as well as the possibility of mapping new samples or cohorts on any of the available landscapes. Below, we describe some test cases to illustrate the utility of our tool, and we also provide a step-by-step tutorial on how to perform basic downstream analyses (available at https://oglandscapes.irbbarcelona.org/tutorial).

**Fig. 1:**
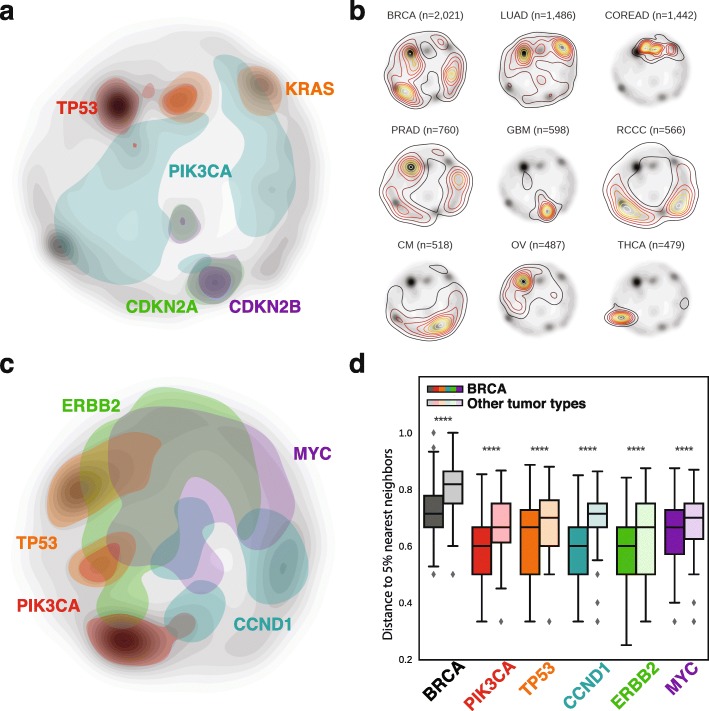
Visual display of the OncoGenomic Landscape of cancer. **a** PanCancer Landscape populated by 15,212 samples of 19 major tumor types of different biological origin (13,827 patients, 434 PDXs, 46 organoids, 905 cell lines). The territories occupied by samples that have at least one of the five most recurrent oncogenic alterations are shaded in different colors and serve as landmarks for molecular interpretation. **b** Distinct territories occupied by the nine most comprehensively characterized tumor types are depicted as transparent level plots overlaid on the PanCancer Landscape background. BRCA breast carcinoma, LUAD lung adenocarcinoma, COREAD colorectal adenocarcinoma, PRAD prostate cancer, GBM glioblastoma multiforme, RCCC renal clear cell carcinoma, CM cutaneous melanoma, OV ovarian cancer, and THCA thyroid cancer. **c** The OncoGenomic Landscape of breast invasive carcinoma (BRCA) patients is shown to illustrate how each of the 19 tumor type-specific landscapes is displayed in our web-server. Colors represent the territories occupied by samples having oncogenic alterations in five breast cancer specific landmark driver genes. **d** Boxplot showing the median distance of breast cancer samples to the 5% nearest neighbors in each comparison. The first two boxes compare the median distance of all breast cancer patients among themselves and to patients with other tumor types. The remaining pairs of boxes focus on patients that have an oncogenic alteration in each of the main five BRCA driver genes. Panels **a**, **b**, and **c** are screenshots directly obtained from the web-server. Panel **d** was generated after performing the statistical analysis outside of the app

Figure 1 displays the distribution of samples across the PanCancer Landscape, including 15,212 genomic profiles from different tissues (see Table 1). As expected, territories corresponding to recurrent drivers such as TP53 or KRAS are well populated (Fig. 1a). Perhaps more interesting is the relatively large amount of patients that occupy a territory shared by TP53 and KRAS alterations, consistent with a significant co-occurrence observed in the MSK-IMPACT Clinical Sequencing Cohort [12, 15], and suggesting a synergistic effect between these alterations. It is also apparent that samples with alterations in CDKN2A and CDKN2B occupy almost identical regions, which agrees with the finding that these two tumor suppressors are usually co-deleted as they are encoded next to each other in a very small locus [22].

Beyond key gene alterations, the PanCancer Landscape retains the tissue of origin of the tumors (Fig. 1b). We can observe how certain tumor types (e.g., glioblastoma or colorectal adenocarcinoma) often present a limited set of driver mutations and are thus restricted to very specific areas in the map, while other types (e.g., breast cancer or prostate adenocarcinoma) show a much more diverse pattern of oncogenic alterations and are widely spread. In both cases, it is possible to cluster cancer patients based on the tissue of origin of their tumor and to identify dominant groups representing each tumor type (Additional file 1: Figure S2), as previously suggested for the 12 major cancer types [3, 4] and, more recently, for the 33 cancer types that comprise the complete TCGA PanCancer Analysis [6]. Moreover, we can zoom in on a region that is specific for a certain tumor type and capture patterns that might otherwise be hidden in the broader PanCancer Landscape (Fig. 1c). For instance, despite their considerable heterogeneity, we see that breast cancer samples are closer to each other than to other tumor types (Fig. 1d). The observed proximity cannot be only attributed to the presence of common driver genes since we observe that tumor samples in different tissues sharing the most frequent driver alterations in breast cancer are significantly more distal. These results strongly suggest that our tumor type-specific territories capture complex mutational signatures that cannot be attained by analyzing driver genes individually.

**Fig. 2:**
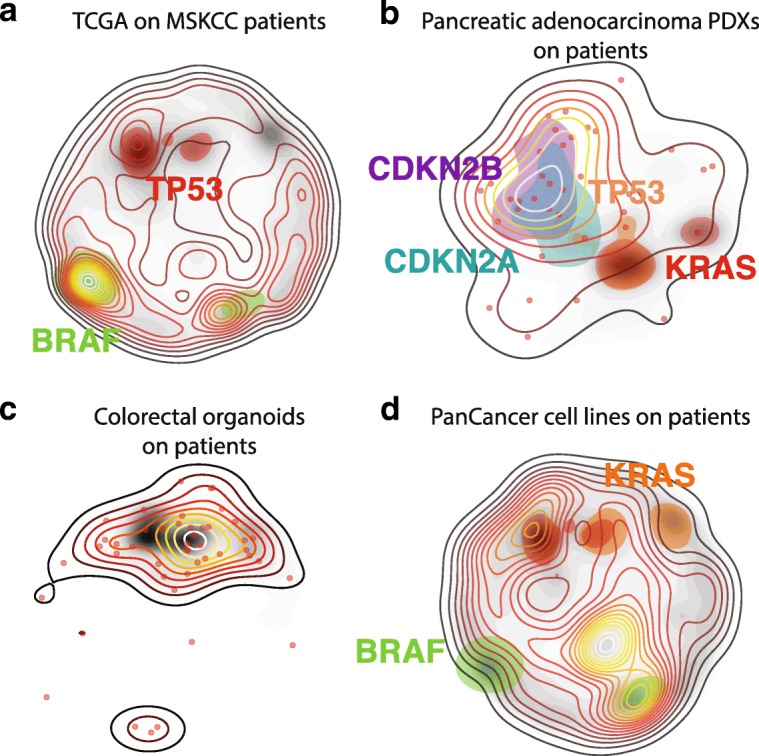
Overlay of different OncoGenomic Landscapes. **a** The cohort of primary tumors from TCGA (*n* = 3850) is displayed as a transparent level plot overlaid on a largest cohort of clinically aggressive tumors from MSKCC (*n* = 9869), represented as a background landscape in gray scale. In a similar way, **b** pancreatic adenocarcinoma PDXs are overlaid on a cohort of PAAD patients (*n* = 377), **c** OncoTrack colorectal organoids (*n* = 46) are overlaid on colorectal adenocarcinoma patients (*n* = 1141), and **d** a panel of 905 cell lines is overlaid on 13,827 PanCancer patients. Panels **b** and **d** are screenshots directly obtained from the web-server. In panels **a** and **d**, we converted one of the landscapes into gray scale to enable a more visual comparison

The accurate comparison of patient or cancer model cohorts is fundamental to evaluate their molecular diversity and, more importantly, to assess whether information such as treatment benefits or prognostic factors learned from a reference group can be safely transferred to a new cohort. For instance, by comparing primary resections of treatment naïve tumors (3850 patients from The Cancer Genome Atlas (TCGA)) to 9869 clinically aggressive tumors from the Memorial Sloan Kettering Cancer Center (MSKCC), we can readily see than alterations in TP53 are much more common in the MSKCC cohort than in TCGA, as recently reported [12], while BRAF alterations show the opposite trend (Fig. 2a). We believe that portrayals like this might also guide the design of clinical basket trials, where patients are selected based on their oncogenomic profiles regardless of their specific tumor type [23].

We can also use OncoGenomic Landscapes to assess the molecular representativity of different model systems (cell lines, organoids, or patient-derived xenografts (PDXs)) with respect to a reference clinical cohort. For example, even though alterations in TP53, KRAS, and CDKN2A are the most prevalent in pancreatic ductal adenocarcinoma patients [22], when we look at the tumors that successfully engrafted in mice (i.e., PDXs), we clearly see that CDKN2A-CDKN2B co-alterations are much more frequent in PDXs than it would be expected from clinical data (Fig. 2b), supporting the idea that the simultaneous inactivation of CDKN2A and CDKN2B is required for the induction of pancreatic cancer in adult mice with overexpressed KRAS^G12D^ and loss of TP53 [22]. Conversely, we observe that the small collection of 69 OncoTrack colorectal organoids [19] spans the molecular diversity seen in a much larger cohort of COREAD patients (188 from TCGA and 953 from MSKCC) (Fig. 2c). Finally, the overlay of 905 cancer cell lines [18] on top of patient samples reveals a lack of cell models to study the effects of KRAS and BRAF mutations alone (Fig. 2d).

**Fig. 3:**
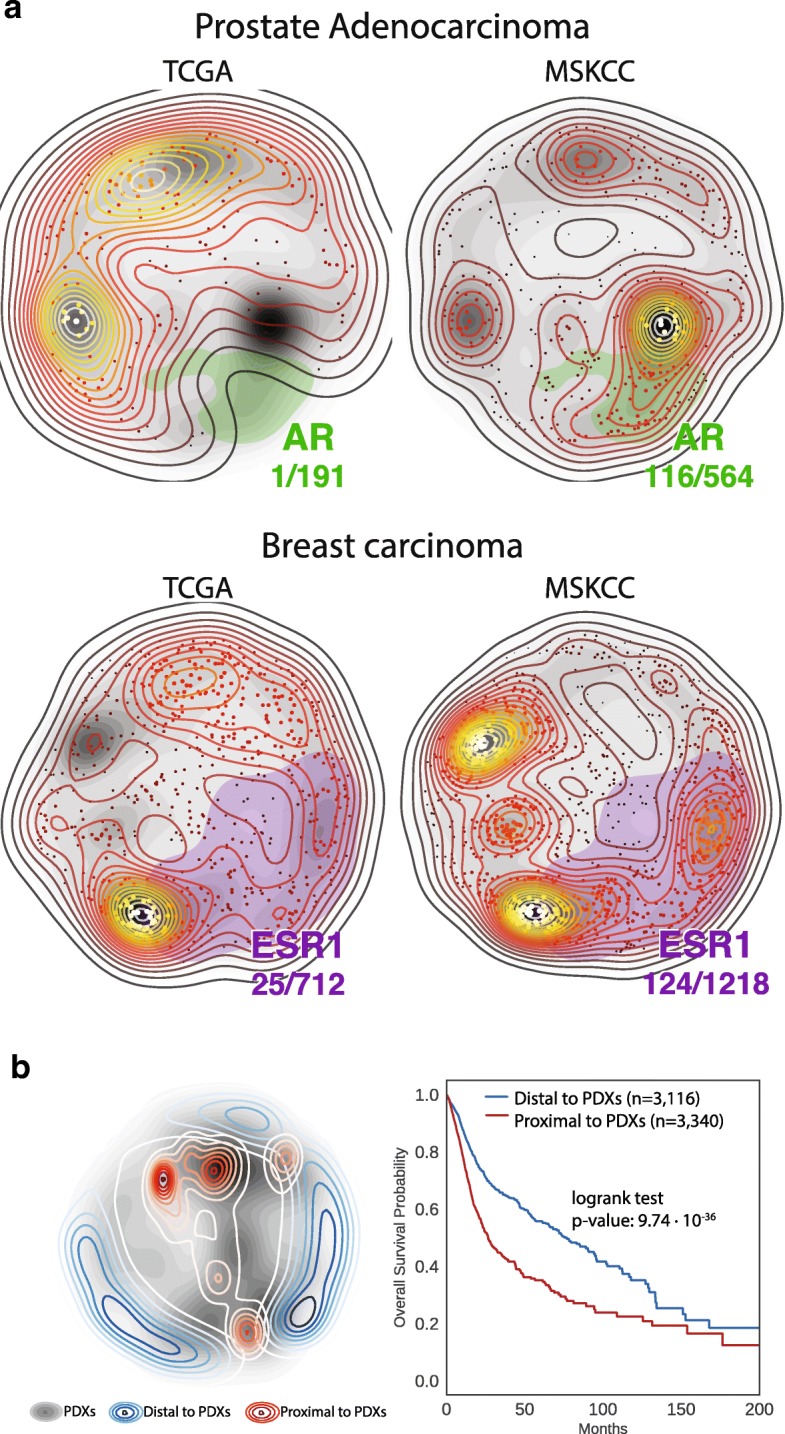
Clinical relevance of OncoGenomic Landscapes. **a** Differences between TCGA and MSKCC cohorts related to resistance to endocrine therapy in PRAD and BRCA. The fraction of patients in each cohort presenting alterations in the androgen receptor (AR) and the estrogen receptor (ESR1) are shown in green and magenta, respectively. **b** Patient distance to PDXs correlates with overall survival probability. The territories occupied by PDXs are shown as a background landscape in gray scale whereas the location of patients that are proximal (red) or distal (blue) to PDXs are shown as transparent level plots. **b** Kaplan-Meyer analysis comparing the overall survival rate of patients that are proximal (red) or distal (blue) to PDXs. Panel **a** is composed of screenshots directly obtained from the webserver. Panel **b** was generated outside the app following the steps described in the tutorial available at https://oglandscapes.irbbarcelona.org/tutorial

Interestingly, we also find that distances in OncoGenomic Landscapes correlate with relevant clinical features. Mutations in the androgen receptor (AR) in prostate and in estrogen receptor (ESR1) in breast cancer are related to acquired resistance to hormonal therapies. The density of patients with mutations in those genes is notoriously higher in MSKCC than in TCGA, consistent with the known clinico-pathological differences of those two cohorts (Fig. 3a). We can also relate territories in the landscape to overall survival probabilities (Fig. 3b). It is well documented that during the establishment of PDXs, there is an engraftment bias towards more aggressive tumors [24, 25]. Accordingly, we see that patients that are proximal to successfully engrafted tumors show a significantly worse prognosis than patients that are distal to PDXs (*p* value 9.74×10^−36^), and the trend remains significant (Cox regression *p* value 2.23 × 10^−12^) after adjusting for possible confounding factors such as tumor type and patient provenance (TCGA or MSKCC). This observation is in line with the recent finding that pancreatic ductal adenocarcinoma patients whose tumors did engraft in mice had significantly shorter recurrence-free and overall survivals than patients whose tumors failed to engraft [24].

## Conclusions

In summary, OncoGenomic Landscapes is a web-based visualization tool that organizes tumor samples, and other cancer models, in a 2D space, enabling the comparison of large cohorts and capturing their molecular heterogeneity. We offer the possibility of mapping new samples and cohorts onto a set of 22 predefined landscapes, providing an intuitive means to visualize user’s data and enrich it with knowledge transferred from the large corpus of cancer samples available today. Contextualizing individual patients in a more general landscape of human cancer is, we believe, a valuable aid for clinical oncologists trying to identify treatment opportunities, maybe in a compassionate use basis, for patients that ran out of standard therapeutic options.

## Availability and requirements

*Project name:* OncoGenomic Landscapes

*Project home page:* https://oglandscapes.irbbarcelona.org

*Operating system(s)***:** Platform independent

*Programming language:* Python, JavaScript *(*Node.js, D3.js and AngularJS)

*Other requirements:* Not applicable

*License:* Not applicable

*Any restrictions to use by non-academics:* Not applicable

## Additional file

Additional file 1: Contains the Supplementary Figures 1–3. (PDF 1055 kb)
