# Supplementary material for: Exploring the OncoGenomic Landscape of cancer
Source: Genome Med. 2018 Aug 3;10:61. doi: 10.1186/s13073-018-0571-0 (PMC6090738; doi:10.1186/s13073-018-0571-0)
Supplement: Supplementary file 1 — Contains the Supplementary Figures 1–3. (PDF 1055 kb) [file 13073_2018_571_MOESM1_ESM.pdf]

Additional file 1

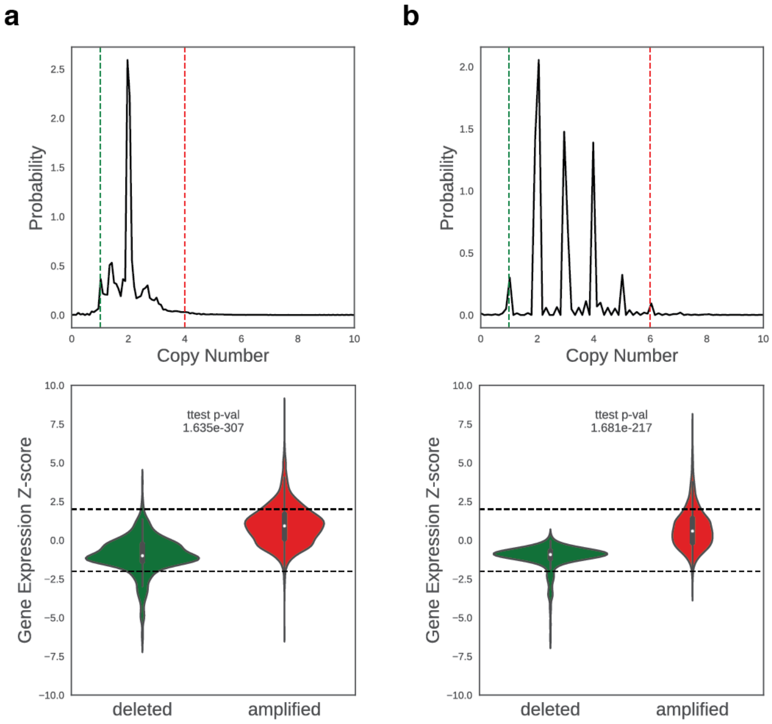

Figure S1. *Copy number alterations in Novartis PDXs (a) and CDSC Cell Line panel (b)*. The top plots show the probability distribution of the estimated absolute copy number per gene in all the samples. Dashed lines indicate the thresholds that were chosen to call deletions (green) or amplifications (red). The bottom plots show the difference in gene expression between deleted tumor suppressors and amplified oncogenes in each dataset.

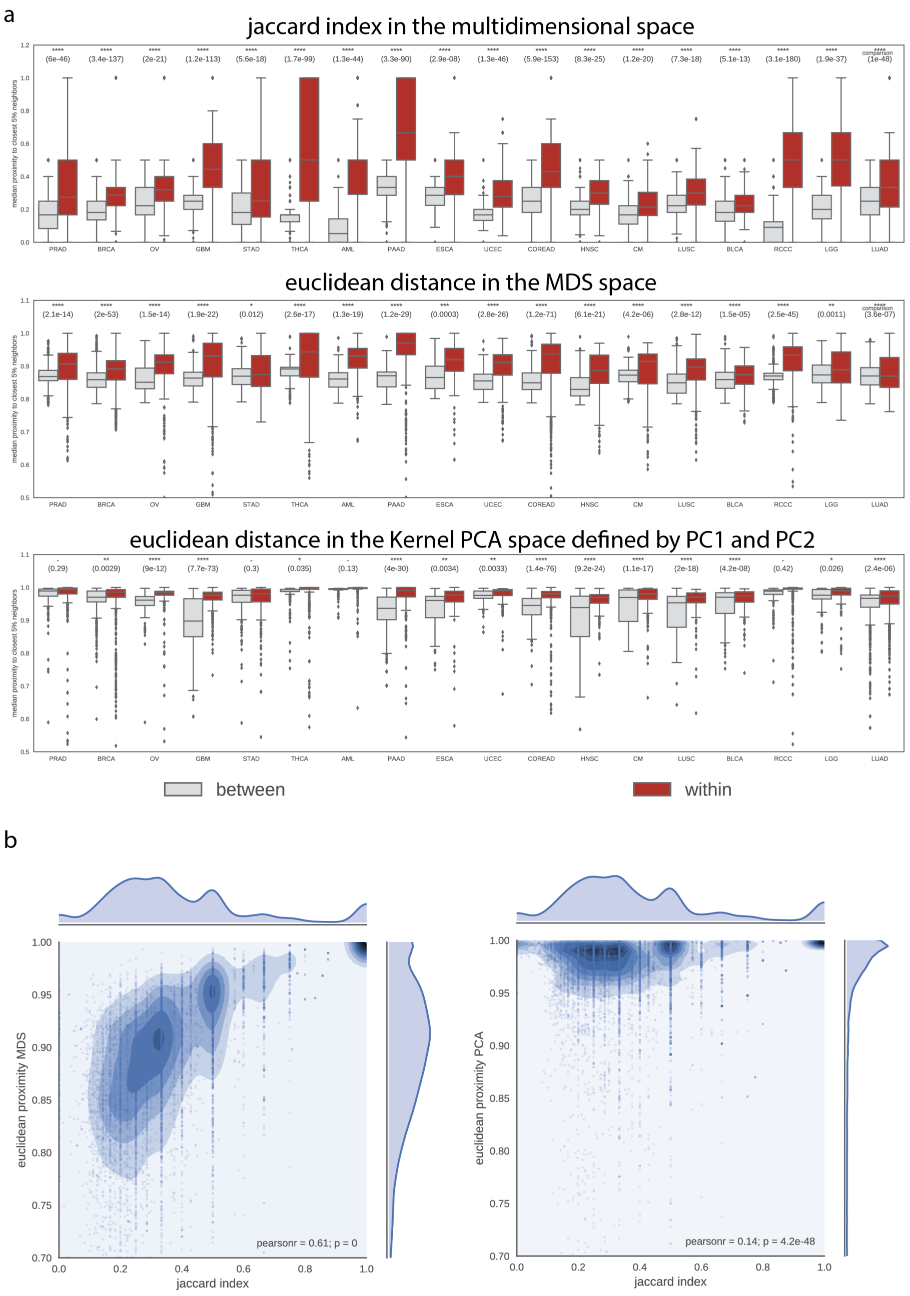

Figure S2. *The organization of samples across the PanCancer landscape reflects the tissue-of-origin of the tumor.* (**a**) Samples of a given tumor type (*within*) are closer to each other in the PanCancer landscape than to samples of other tumor types (*between*) when measuring the median Jaccard similarity coefficient with respect to the closest 5% neighbors in the multidimensional space. The clustering of samples based on tissue-of-origin is also significant when measuring the median Euclidean proximity to the closest 5% neighbors in the two-dimensional MDS space and in the space defined by the first two components of a Kernel Principal Component Analysis (Kernel PCA). (**b**) Correlation between the proximity of each sample to the closest 5% samples of the same tumor type in the MDS space (left) or in the Kernel PCA (right) and the proximity to the closest 5% samples of the same tumor type in the multidimensional space (Jaccard index).

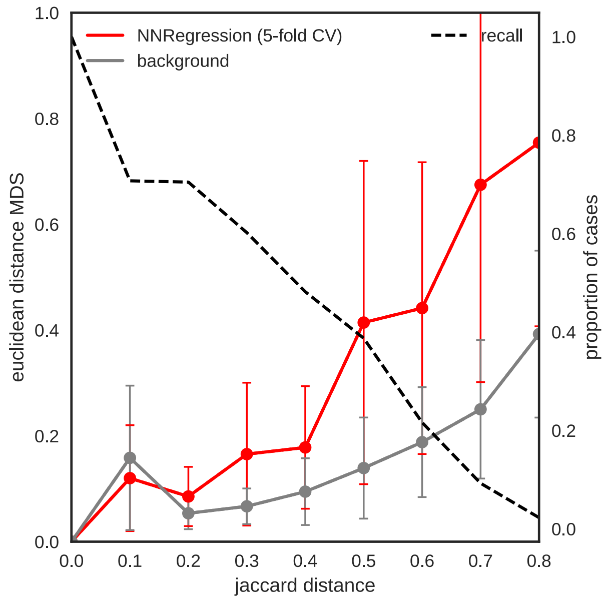

Figure S3. Comp*arison between the mapping errors associated to the nearest neighbor search to the intrinsic error of SMACOF MDS.* The red line represent the performance of the Nearest Neighbor Regressor in a five-fold corss-validation exercise, in terms of Euclidean distance between the predicted and the observed coordinates in the MDS space with respect to the Jaccard distance between the test sample and its closest neighbor. The gray line represents the variablitiy in the SMACOF MDS projections of pairs of samples, measured as Euclidean distance in the MDS space, with respect to their Jaccard distance in the n-dimensional space. Solid lines represent the median distance, with error bars indicating its median absolute deviation. The dashed line represents the cumulative proportion of cases in the test set that are at a given distance or greater from its nearest neighbor.
